# MYC regulates metabolism through vesicular transfer of glycolytic kinases

**DOI:** 10.1098/rsob.210276

**Published:** 2021-12-01

**Authors:** Alexia Tsakaneli, Victor Corasolla Carregari, Martina Morini, Alessandra Eva, Giuliana Cangemi, Olesya Chayka, Evgeny Makarov, Sandra Bibbò, Emily Capone, Gianluca Sala, Vincenzo De Laurenzi, Evon Poon, Louis Chesler, Luisa Pieroni, Martin R. Larsen, Giuseppe Palmisano, Arturo Sala

**Affiliations:** ^1^ Institute of Environment, Health and Societies, Department of Life Sciences, Brunel University London, UB8 3PH Uxbridge, UK; ^2^ GlycoProteomics Laboratory, Department of Parasitology, Institute of Biomedical Sciences, University of Sao Paulo, Av. Prof. Lineu Prestes, 1374 Sao Paulo, Brazil; ^3^ Department of Experimental Neuroscience, Proteomics and Metabonomics Unit, Fondazione Santa Lucia-IRCCS, Rome, Italy; ^4^ Laboratorio di Biologia Molecolare, IRCCS Istituto G. Gaslini, Genoa, Italy; ^5^ Laboratorio di Analisi, IRCCS Istituto G. Gaslini, Genoa, Italy; ^6^ Department of Innovative Technologies in Medicine and Dentistry, University of Chieti-Pescara, Center for Advanced Studies and Technology (CAST) Chieti, Italy; ^7^ Division of Clinical Studies and Cancer Therapeutics, The Institute of Cancer Research, London, UK; ^8^ Department of Biochemistry and Molecular Biology, University of Southern Denmark, Odense, Denmark

**Keywords:** extracellular vesicles, MYC, MYCN, neuroblastoma, Warburg effect

## Abstract

Amplification of the proto-oncogene *MYCN* is a key molecular aberration in high-risk neuroblastoma and predictive of poor outcome in this childhood malignancy. We investigated the role of MYCN in regulating the protein cargo of extracellular vesicles (EVs) secreted by tumour cells that can be internalized by recipient cells with functional consequences. Using a switchable MYCN system coupled to mass spectrometry analysis, we found that MYCN regulates distinct sets of proteins in the EVs secreted by neuroblastoma cells. EVs produced by MYCN-expressing cells or isolated from neuroblastoma patients induced the Warburg effect, proliferation and c-MYC expression in target cells. Mechanistically, we linked the cancer-promoting activity of EVs to the glycolytic kinase pyruvate kinase M2 (PKM2) that was enriched in EVs secreted by MYC-expressing neuroblastoma cells. Importantly, the glycolytic enzymes PKM2 and hexokinase II were detected in the EVs circulating in the bloodstream of neuroblastoma patients, but not in those of non-cancer children. We conclude that MYC-activated cancers might spread oncogenic signals to remote body locations through EVs.

## Graphical abstract

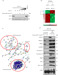

## 1. Introduction

Neuroblastoma is a childhood malignancy originating from the peripheral nervous system that is still a leading cause of oncological death. About 50% of high-risk neuroblastomas are *MYCN* amplified and a causational link between MYCN activation in the nervous system and tumourigenesis has been firmly established [1,2]. The product of the *MYCN* amplicon is a transcription factor belonging to the MYC family of oncoproteins, frequently activated in neuroblastoma and other childhood malignancies [3].

In previous studies, it was shown that cancer cells expressing MYC proteins modify the tumour microenvironment via the activation or inactivation of growth factors, cytokines and immune checkpoint regulators [4–6]. The hypothesis that MYC expression could induce non-cell autonomous effects is also supported by evidence of heterogeneous (focal) amplification of *MYCN* in neuroblastoma tumours. Focally amplified neuroblastomas have a negative prognosis, indistinguishable from uniformly amplified cases, suggesting that MYCN could promote a cross-talk between parts of the cancer with a different amplification status [7]. We therefore hypothesized that MYCN could promote neuroblastoma growth by regulating the secretion or content of extracellular vesicles (EVs) which would modify the metabolic activity of recipient cells. EVs are formed by exosomes, of endocytic origin, and microvesicles, produced by membrane budding, that change the behaviour of recipient cells by the delivery of proteins and nucleic acids [8]. Cancer-produced EVs play a key role in the metastatic dissemination and/or pro-angiogenic activity of solid tumours [9]. While the pro-tumourigenic or pro-metastatic role of EVs has been highlighted in different types of adult cancers [10–16] it is not known whether they play a role in the context of *MYCN*-amplified neuroblastoma.

Using neuroblastoma as a model, we have therefore investigated the hypothesis that MYC-activated tumours could spread oncogenic signals to other body and tissue compartments via regulation of EVs protein cargo.

## 2. Material and methods

### 2.1. Clinical material

Plasma samples (10 NB patients with *MYCN* amplification and 10 neuroblastoma patients without *MYCN* amplification) were provided by the BIT-Gaslini Biobank of the IRCCS G. Gaslini Institute in Genova (Italy). The use of human biological specimens stored in the BIT-Gaslini Biobank was approved by the Ethical Committee of the Gaslini Institute. Plasma was collected by centrifuging blood at 1 200 x g for 10 min at room temperature and immediately stored at −80°C. Twenty age-matched plasma samples were obtained from leftover plasma after routine clinical analyses from outpatients. A written consent allowing the collection of samples and the use of clinical and non-genetic data for clinical research was signed by patients' guardians.

### 2.2. Cell culture, plasmid and siRNA transfections

The human neuroblastoma cell lines SH-EP and KELLY were purchased from the ATCC. TET21-N were kindly supplied by Prof Giovanni Perini. Neuroblastoma cells were cultured in DMEM supplemented with 10% FBS and 100 units ml^−1^ Penicillin/Streptomycin. TET21-N cells were routinely maintained in medium containing 0.2 mg ml^−1^ G-418 (11811031, GIBCO) and 0.15 mg ml^−1^ Hygromycin B (10453982, Invitrogen), and to switch off MYCN expression cells were cultured in the presence of 1 µg ml^−1^ doxycycline (D9891, Sigma-Aldrich). For EV preparation, cells were cultured in microvesicles depleted medium prepared as follows. In brief, DMEM supplemented with 20% FBS was ultra-centrifuged at 100 000 x g for 20 h. The pellet was discarded, the medium was diluted 1 : 2 with fresh DMEM and used. TET21-N cells were transfected using the transfection reagent jetPrime (114–01, Polyplus transfection) with 50 µM siRNAs targeting pyruvate kinase M2 (PKM2) (SASI_Hs01_00217689, EHU147561), Rab27a (EHU091501) or rLuc as a control (EHURLUC) purchased from Sigma-Aldrich. Forty-eight hours after transfection, the cells were collected and equal numbers were seeded on tissue culture inserts with 0.4 µm pores (83.3932.040, Sarstedt) for co-culture with SH-EP cells. The remaining cells were used for protein analysis. CD63-pEGFP C2 (62964) and pEGFP-C1-PKM2 (64698) plasmids were purchased from Addgene and transfected using the transfection reagent jetPrime.

### 2.3. Extracellular vesicle isolation and quantification

Cell supernatants were centrifuged at 3000 x g for 30 min and 100 000 x g for 70 min at 4°C. The pellet was resuspended in PBS and ultra-centrifuged at 100 000 x g for 70 min. The concentration of EV proteins was quantified with the bicinchoninic acid (BCA) protein assay (23225, Pierce). For EVs preparations from patients, 500 µl of frozen plasmas were processed using the Qiagen ExoEasy Maxi EVs isolation kit (76064, Qiagen). EVs were enumerated by lipophilic cationic dye staining and polychromatic flow cytometry, as described [17].

### 2.4. Transmission electron microscopy

EVs resuspended in PBS were fixed by mixing them in an equal volume of 4% paraformaldehyde (PFA). Glutaraldehyde was added to reach a final concentration of 1%. For negative staining, the fixed EVs were mixed with 1% sodium phosphotungstate (K-PTA), and a drop of this mixture was placed onto a carbon-coated electron microscope grid, glow discharged for 20 s. The mixture was left on the grid for 1 min and then removed almost completely with a filter paper.

### 2.5. Nanoparticle tracking analysis

The size distribution of EVs was quantified with a NanoSight N300 using a 640 nm laser (Malvern Instruments, Malvern). The samples were injected in the NanoSight sample cubicle and the data were analysed with the NanoSight NTA3.2 software.

### 2.6. PKH67 staining and Imagestream analysis

For the membrane staining of the EVs, the PKH67 green fluorescent cell linker mini kit (MINI67, Sigma-Aldrich) was used. Briefly, EVs pellets were resuspended in 200 µl diluent C; 0.8 µl of the PKH67 dye were added to 200 µl of diluent C and mixed with the resuspended EVs and incubated at room temperature for 4 min. The reaction was stopped with an equal volume (400 µl) of 1% BSA in PBS. The EVs were ultra-centrifuged at 100 000 x g at 4°C for 70 min and the pellet resuspended in PBS. The stained EVs were visualized with ImageStream flow cytometry. All samples were acquired on an ImageStreamX Mk II imaging cytometer, X60 magnification with low flow rate/high sensitivity without EDF using INSPIRE software. Channel 01 was set for bright field, channel 06 as a scattering channel and channel 02 for the required fluorescence channel (laser 488).

### 2.7. Proteomic analysis

The EVs preparations were resuspended in RIPA buffer (150 mM NaCl, 1 mM EDTA, 0.5% sodium deoxycolate, 0.1% SDS, Sigma) with freshly added protease and phosphatase inhibitors (Protease Inhibitor Cocktail, Sigma). After 30 min of mild sonication to extract proteins attached to the EV membranes, protein content was quantified by the Bradford method (BIO-RAD). Three aliquots of protein extracts from each condition (20 µg) were transferred onto Microcon-10 Centrifugal Filter (cut-off 10 kDa) for FASP protein digestion [18]. Proteins were reduced by 8 mM DTT and alkylated using 0.05 M iodacetamide. Each sample was then digested with trypsin overnight, after which digestions were blocked by adding formic acid to a final concentration of 0.2% (v/v). Peptides were recovered in 0.05 M AMBIC-SDS, concentrated in a speedvac and stored at −80°C until use. The digested peptides were loaded onto a Symmetry C18 (5 µm, 180 µm × 20 mm) precolumn (Waters Corp.) and subsequently separated by a 120 min reversed phase gradient at 300 nl min^−1^ (linear gradient, 2–85% ACN over 120 min) using a HSS T3 C18 (1.8 µm, 75 µm × 150 mm) nanoscale LC column (Waters Corp.) maintained at 40°C. The peptides were analysed by a High Definition Synapt G2-Si mass spectrometer directly coupled to the chromatographic system. Differential protein expression was evaluated with a data-independent acquisition of shotgun proteomics analysis by expression configuration mode (MSe). The mass spectrometer operated in ‘expression mode’ switching between low (4 eV) and high (15–40 eV) collision energies on the gas cell, using a scan time of 1.5 s per function over 50–2000 *m/z*. All spectra were acquired in ion mobility mode by applying a wave velocity for the ion separation of 900 m s^−1^ and a transfer wave velocity of 175 m s^−1^. The processing of low and elevated energy, added to the data of the reference lock mass ([Glu1]-Fibrinopeptide B Standard, Waters Corp) provided a time-aligned inventory of accurate mass-retention time components for both the low and elevated energy. Each sample was run in three technical replicates.

The analysis of differentially expressed proteins was performed according to Silva *et al.* [19] and Visser *et al.* [20]. Continuum LC-MS data from three replicates for each sample was processed for qualitative and quantitative analysis using the ProteinLynx Global Server v. 3.0.2 software (PLGS, Waters Corp.). Proteins were identified by searching the Homo Sapiens (UniProt KB/Swiss-Prot Protein reviewed) and ExoCarta databases to which sequence from *Saccharomyces cerevisiae* Enolase (UniProtKB/Swiss-Prot AC: P00924) was appended in order to perform the quantitative analysis. Search parameters were set as: automatic tolerance for precursor ions and for product ions, minimum three fragment ions matched per peptide, minimum three fragment ions matched per protein, minimum two peptides matched per protein, one missed cleavage, carbamydo methylation of cysteines and oxidation of methionines as fixed and variable modifications, false-positive rate of the identification algorithm under 1% and 100 fmol of the enolase set as calibration protein concentration. The expression analysis was performed considering technical replicates available for each experimental condition (i.e. untreated and treated with doxycycline) following the hypothesis that each group is an independent variable. The protein identifications were based on the detection of more than two fragment ions per peptide, more than two peptides measured per protein. The list of normalized proteins was screened according to the following criteria: protein identified in at least two out of three runs of the same sample with a fold change of regulation higher than ±20%; only modulated proteins with a *p* < 0.05 were considered significant.

A different EV preparation was re-suspended in lysis buffer (7 M urea, 2 M thiourea and protease inhibitors). Proteins were reduced with 10 mM DTT (Sigma-Aldrich) for 30 min at room temperature, alkylated with 40 mM iodoacetamide (Sigma-Aldrich) for 30 min in the dark and digested with trypsin (Promega) in the ratio 1 : 50 (μg trypsin : μg protein) in 50 mM ammonium bicarbonate solution at 30°C overnight. The reaction was stopped with 1% formic acid and then the sample was desalted with C18 columns (StageTips). Three biological replicates were prepared for each condition. Peptides were separated by Reprosil-Pur C18-AQ column (3 μm; Dr. Maisch GmbH, Germany) using Easy nano-LC HPLC (Proxeon, Odense, Denmark). The HPLC gradient was 0–34% B solvent (A = 0.1% formic acid; B = 90% ACN, 0.1% formic acid) in 70 min at a flow of 250 nl min^−1^ for a total of 93 min run. The MS analysis was performed using the LTQ-Orbitrap Velos (Thermo Scientific, Bremen, Germany). The mass range was 350–1500 *m/z* at a resolution of 60 000 at 400 *m/z*. For each MS scan, collision-induced dissociation (CID) fragmentation was performed on the 15 most intense ions in the linear ion trap. The parameters for data acquisition were activation time = 5 ms, normalized energy = 35, Q-activation = 0.25, exclusion = available with repeat count 1, exclusion duration = 30 s and intensity threshold = 5000. The mass spectrometry proteomics data have been deposited to the ProteomeXchange Consortium via the PRIDE repository on https://www.ebi.ac.uk/pride/. The raw files were searched using the MaxQuant version 1.2.7.429 and the MS/MS spectra were searched using the Andromeda search engine against the Uniprot-reviewed Human Protein Database. The initial maximal allowed mass tolerance was set to 20 ppm for precursor and then set to 4.5 ppm in the main search and to 0.5 Da for fragment ions. Enzyme specificity was set to trypsin with a maximum of two missed cleavages. Carbamidomethylation of cysteine (57.02 Da) was set as a fixed modification, and oxidation of methionine (15.99 Da) and protein N-terminal acetylation (42.01 Da) were selected as variable modifications. Bioinformatics analysis was performed using the software Perseus v. 1.5.2.6 available in the MaxQuant environment and reverse and contaminant entries were excluded from further analysis. Protein FDR was calculated in the MaxQuant software and kept below 1%. Label-free quantification intensity values were considered to relatively compare the abundance of proteins present in the two conditions and regulated proteins were identified with statistical t-test with *p*-value < 0.05 with Benjamini–Hochberg correction.

### 2.8. Western blot analysis

Equal amounts of proteins were separated by SDS-PAGE electrophoresis, transferred onto nitrocellulose membranes which were blocked with 5% milk in TBS with 0.1% Tween-20 for 1 h at room temperature. The membranes were incubated over night at 4°C with primary antibodies against CD63 (1 : 200, sc-5275 Santa Cruz), eukaryotic elongation factor 2 (eEF2) (1 : 1 000, 2332, Cell Signalling), glyceraldehyde 3-phosphate dehydrogenase (GAPDH) (1 : 1000, 5174S, Cell Signalling), Hexokinase I (1 : 1000, C35C4, Cell Signalling), Hexokinase II (1 : 100, C64G5, Cell Signalling), MYCN (1 : 200, sc-53993, Santa Cruz), phosphofructokinase PFKP (1 : 1 000, 8164S, Cell Signalling), PKM1/2 (1 : 1 000, 3190S, Cell Signalling), PKM2 (1 : 1 000, 4053S, Cell Signalling), rib. L10a (1 : 500, sc-100827, Santa Cruz) and β-actin (1 : 1000, 3700, Cell Signalling). After washing with TBS 0.1% Tween-20 three times for 5 min at room temperature, the membranes were incubated with horseradish peroxidase (HRP)—conjugated secondary antibodies for 1 h at room temperature (anti-mouse-HRP, 1 : 10 000, sc-2031, Santa Cruz, anti-rabbit-HRP, 1 : 10 000, sc-2313, Santa Cruz). The membranes were washed three times for 5 min at room temperature with TBS 0.1% Tween-20 and incubated with enhanced chemiluminescent substrate (32106, Pierce). The protein bands were visualized with X-ray films.

### 2.9. Cell counts and MTS proliferation assays

2 × 10^3^ SH-EP cells were plated into each well of 96-well plates in the presence or absence of EVs that were replenished every day (10 µg of EV proteins per well). Cells were harvested at 24, 48 or 72 h and counted with a haemocytometer. For MTS assays, 20 µl of MTS/PMS solution (G3582, Promega) were added to each well and incubated for 4 h. Absorbance at 490 nm was measured with a spectrophotometer and expressed as optical density units.

### 2.10. Glycolysis assays

2 × 10^3^ SH-EP cells were seeded in each well of 96-well plates and incubated overnight in a 5% CO_2_-humidified incubator at 37°C. The next morning, EVs were added to the cells (10 µg of EV proteins per well). After 24 h, the production of L-lactate was evaluated with the glycolysis cell-based assay kit (600450, Cayman Chemical). Briefly, the cell supernatants were centrifuged at 1 200 rpm for 5 min; 90 µl of assay buffer were transferred to each well of a 96-well plate plus 10 µl of cell supernatants or 10 µl of L-lactate solution used as a positive control. A 100 µl of reaction solution was added to each well and the mix was incubated for 30 min at room temperature with gentle shaking. The absorbance was measured at 490 nm using a spectrophotometer. For the SH-SY5Y cells, the consumption rate of glucose was quantified using a Glucose Uptake Colorimetric Assay Kit (Sigma-Aldrich, MAK083) according to manufacturer's instructions.

### 2.11. Seahorse analysis

Mitochondrial bioenergetics assays were carried out using an XFe96 analyser from Agilent Seahorse Bioscience (Santa Clara) following published protocols [21]. The XF assay medium (HCO_3_-free modified DMEM, Seahorse Bioscience) was supplemented with 10 mM glucose, 2 mM l-glutamine and 1 mM sodium pyruvate. 15 × 10^3^ cells were grown in each well of Seahorse assay plates. After 4 h, at the step of Seahorse medium exchange, 100 µg ml^−1^ of EVs from MYCN-positive or -negative TET21-N cells or PBS were added and the cells where incubated for 20 h. The mitochondrial respiration test was performed by sequential addition of μM oligomycin, 1.5 µM FCCP and 1 µM rotenone/antimycin A to the cells. Using the XF cell Mitostress test kit (103015–100; Seahorse Bioscience), we examined the following mitochondrial parameters: basal mitochondrial respiration (basal cellular respiration minus non-mitochondrial respiration), maximal respiratory capacity (maximal uncoupled respiration minus non-mitochondrial respiration) and non-mitochondrial respiration (rotenone/antimycin A-inhibited respiration).

### 2.12. Immunofluorescence

The cells were cultured on coverslips or in microwells (154534, Thermo Scientific). The cells were fixed in 4% PFA for 10 min at room temperature. After washing with PBS for three times, the cells were permeabilized with 0.5% Triton X-100 in PBS for 10 min at room temperature. After washing with PBS for three times, cells were blocked with 5% BSA in PBS for 30 min at 37°C. The cells were incubated with the primary antibody against *p*-H3 (T11) (1 : 100, ab5168, Abcam) or cMYC (1 : 800, 5605, Cell Signalling) diluted in 3% BSA in PBS for 30 min at 37°C. After washing with PBS for three times, the cells were incubated with anti-rabbit FITC secondary antibody (711-095-152, Jackson ImmunoResearch) diluted 1 : 200 in BSA 1% in PBS for 30 min at 37°C. One drop of Vectashield antifade mounting medium, containing DAPI, (H-1200, Vector Laboratories) was added to each sample before analysis. Fiji software was used for the quantification of the intensity.

### 2.13. Immunohistochemistry

Tumour sections were incubated in HistoChoice clearing agent (H2779, Sigma Aldrich) to remove the wax and then sequentially in 100%, 90%, 70% and 50% ethanol. In the final step, the sections were incubated in dH_2_O twice for 5 min. Antigens were unmasked by boiling the sections in citrate solution in a microwave oven. Endogenous peroxidase was removed with incubation in 3% H_2_O_2_ (216763, Sigma Aldrich) in distilled H_2_O for 20 min at RT. The sections were next incubated with mouse on mouse (MOM) mouse IgG blocking reagent (BMK-2202, Vector Laboratories) for 1 h at room temperature. After blocking, the sections were washed in PBS, incubated for 5 min in MOM diluent (BMK-2202, Vector Laboratories), the excess of diluent was removed and the sections were incubated with mouse anti-MYCN (1 : 100, OP13, Calbiochem) or anti-c-MYC (1 : 50, sc-40, Santa Cruz) primary antibodies diluted in blocking buffer 1 : 10 and incubated overnight at 4°C. The sections were washed in PBS 1X twice for 2 min and then incubated with biotinylated anti-mouse IgG reagent (BMK-2202, Vector Laboratories) for 10 min followed by incubation with Avidin, NeutrAvidin HRP conjugate (A2664, Molecular Probes) diluted 1 : 1000 in PBS for 2 h at room temperature. One drop of ImmPACT DAB chromogen (SK-4105, Vector Laboratories) was diluted in 1 ml of ImmPACT DAB diluent and applied on the sections for 2–10 min until desired colour intensity was developed. After staining with hematoxylin (H3136, Sigma Aldrich), the sections were dehydrated and mounted with a glass coverslip using one drop of DPX mountant (O6522, Sigma Aldrich).

### 2.14. Statistical analysis

All data are expressed as means ± s.e.m. Statistical tests used were comparison of means with two-tailed paired samples or independent samples t-test, as indicated in the figure legends; *p*-values less than 0.05 were considered significant. All experiments were replicated independently and the number of replicates is indicated in the figure legends. All statistical tests were performed with the IBM SPSS software.

## 3. Results

### 3.1. Extracellular vesicles secreted by neuroblastoma cells with activated MYCN are enriched in oncogenic glycolytic enzymes

**Figure 1.**
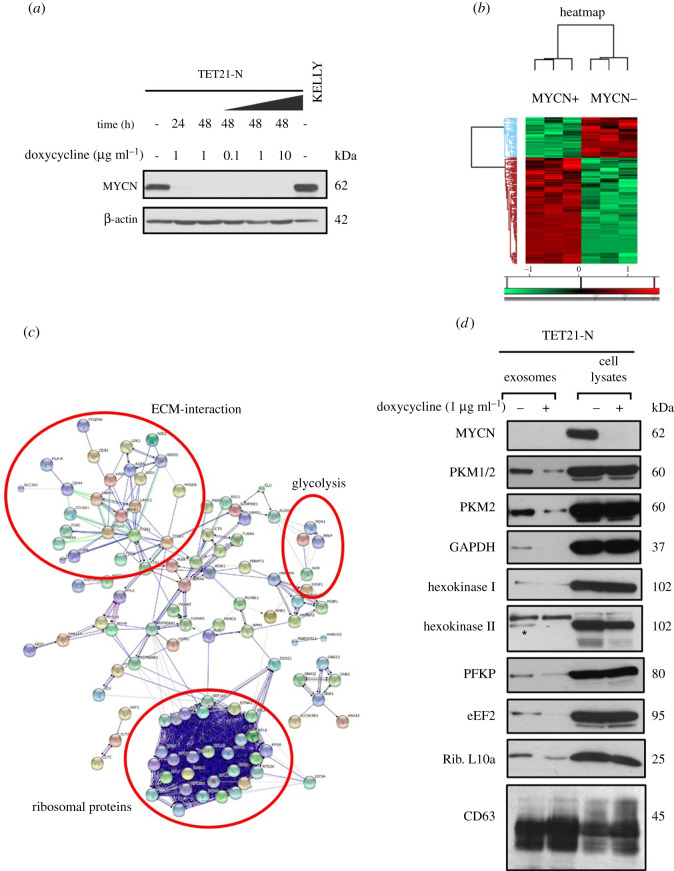
MYCN expression regulates EVs protein cargo in neuroblastoma cells. (*a*) Western blot analysis demonstrating the conditional expression of MYCN in TET21-N cells in the presence (MYCN-off) or absence (MYCN-on) of doxycycline. Protein lysates from the *MYCN*-amplified cell line KELLY were used as a positive control. β-actin was used as a loading control. (*b*) Heat map analysis showing a drastic difference in protein expression—either upregulated (red) or downregulated (green)—in EVs secreted by TET21-N cells expressing or non-expressing MYCN. Mass spectrometry analysis was conducted in triplicate samples (biological replicates). (*c*) Pathway analysis of the proteins identified in EVs from MYCN-positive and -negative TET21-N cells. The protein groups enriched in the MYCN-positive condition are evidenced by the red circles. (*d*) Western blot analysis of EVs and total cell lysates isolated from the TET21-N cells. The EV marker CD63 was used as a loading control. An asterisk indicates the hexokinase II protein band at 105 kD, whereas the 125 kD band enriched in vesicles is of spurious nature.

The relationship between *MYCN* expression and EV protein cargo was investigated using the TET21-N neuroblastoma cell line. TET21-N have been stably transfected with a *MYCN*, doxycycline-repressible and expression vector [22]; in the absence of doxycycline, MYCN protein is constitutively expressed (figure 1*a*). The supernatant of TET21-N cells, expressing or non-expressing MYCN, was collected and EVs were purified by differential ultracentrifugation. Nanoparticle tracking analysis and transmission electron microscopy confirmed the presence of exosome-like EVs with an average diameter of 100–200 nm (electronic supplementary material, figure S1A, B). The membrane integrity of the vesicles preparations was validated by staining with the membrane-specific dye PKH67 and observing the EVs with ImageStream flow cytometry (electronic supplementary material, figure S1C). The classical EV marker CD63 was used to validate the vesicles preparation (electronic supplementary material, figure S1D). Subsequently, a label-free quantitative mass spectrometry-based proteomic analysis was performed on EVs isolated from TET21-N cells expressing or non-expressing MYCN. In total, 152 EV proteins were differentially regulated between the two conditions with 111 proteins upregulated and 41 proteins downregulated upon MYCN expression (figure 1*b*; electronic supplementary material, Dataset S1). Pathway analysis suggests that glycolytic enzymes, ribosomal and extracellular matrix (ECM) interaction proteins were mostly enriched in the EVs secreted by the MYCN-positive cells (figure 1*c*). The differential expression of proteins implicated in glycolysis was validated by western blotting. PKM2, hexokinase I and hexokinase II were upregulated in the EVs secreted by TET21-N cells expressing MYCN compared to the condition in which *MYCN* gene expression was switched off. Other proteins linked to metabolism such as eEF2, PFKP, GAPDH and ribosomal protein L10a were also confirmed to be enriched in the EVs secreted by MYCN-positive neuroblastoma cells (figure 1*d*). The expression levels of vesicular proteins did not reflect those observed in the corresponding cellular lysates (figure 1*d*), indicating that MYCN might control their loading into EVs.

### 3.2. Vesicles circulating in the plasma of neuroblastoma patients contain pyruvate kinase M2 and hexokinase II

**Figure 2.**
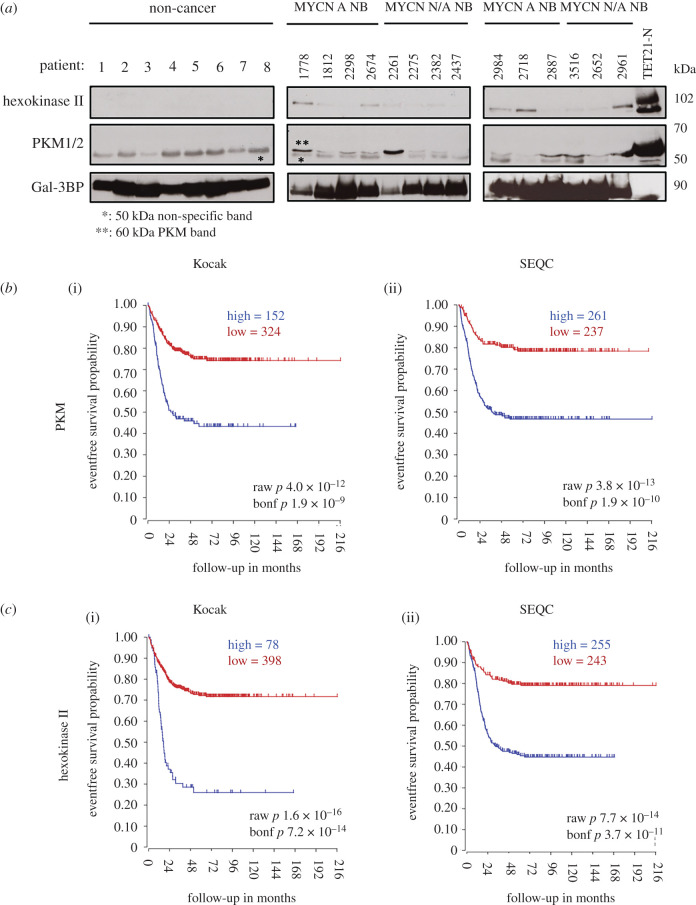
Analysis of vesicular proteins in the plasma of neuroblastoma patients. (*a*) The indicated proteins were identified in the cargo of EVs isolated from the plasma of patients with (MYCN A NB) or without (MYCN N/A NB) *MYCN*-amplified neuroblastomas by western blot analysis. Plasmas from non-cancer patients were used as controls. Gal-3BP was used as loading control and total cell lysates from TET21-N cells were used as a positive control for PKM and hexokinase II. * indicates non-specific band; ** indicates specific band. Kaplan–Meier survival curve correlating the expression of PKM (*b*) and hexokinase II (*c*) with event-free neuroblastoma patients' survival in the Kocak (i) and SEQC (ii) datasets available in the R2 genomic visualization platform (https://hgserver1.amc.nl/cgi-bin/r2/main.cgi).

Since PKM2 and hexokinase II are glycolytic enzymes with oncogenic potential [23], detectable in the EVs of neuroblastoma cells, we wondered whether we could also detect glycolytic enzymes in the EVs circulating in the plasma of patients bearing neuroblastomas. We therefore isolated EVs from the plasmas of neuroblastoma or non-cancer patients, which were used as controls. We detected vesicles of sizes similar to those detected in the supernatant of neuroblastoma cells, which were positive to the CD63 marker (electronic supplementary material, figure S2A, B). To further validate the plasma vesicles preparations, we used an antibody against galectin-3-binding protein (Gal-3BP), which was shown to detect exosomes secreted by neuroblastoma cells [24] (electronic supplementary material, figure S2C). Remarkably, PKM2 and hexokinase II were detected in the EVs isolated from plasma samples of neuroblastoma patients, but were completely absent in the vesicles of non-cancer patients (figure 2*a*). The expression of the enzymes was similar in vesicles preparations from patients with *MYCN-*amplified or non-amplified tumours. However, *MYCN* non-amplified tumours often present high levels of expression of *MYC* (c-MYC) [25], which might explain why PKM2 and hexokinase II levels were similar in vesicles from the two groups of patients. We determined that the prominent 50 kD protein band detected in all plasma samples is of spurious nature (electronic supplementary material, figure S2D). The expression of the *PKM* and *HK2* genes strongly correlates with poor patient prognosis, suggesting that their products might confer a growth or survival advantage to neuroblastoma cells (figure 2*b,c*). We analysed EVs secreted from multiple neuroblastoma cell lines to confirm that glycolytic enzymes with oncogenic potential were enriched in MYC-expressing cells. EVs or total cell lysates were prepared from *MYCN*-amplified (LAN5, Kelly) and non-*MYCN*-amplified (SH-SY5Y, SH-EP and GIMEN) neuroblastoma cell lines. The *MYCN* non-amplified cell lines, as expected, did not display detectable levels of MYCN protein. However, SH-SY5Y and SH-EP expressed high levels of MYC (c-MYC), whereas GIMEN cells did not express either MYC family members (electronic supplementary material, figure S3A). Consistent with the hypothesis that MYC family members regulate EV protein cargo, vesicles derived from MYC/MYCN-positive cell lines were enriched for the glycolytic enzymes hexokinase II and PKM2 in comparison to MYC-negative GIMEN cells (electronic supplementary material, figure S3B).

### 3.3. Extracellular vesicles secreted by MYCN-positive cells induce glycolysis, increase respiration capacity, ATP production and proliferation of recipient cells

**Figure 3.**
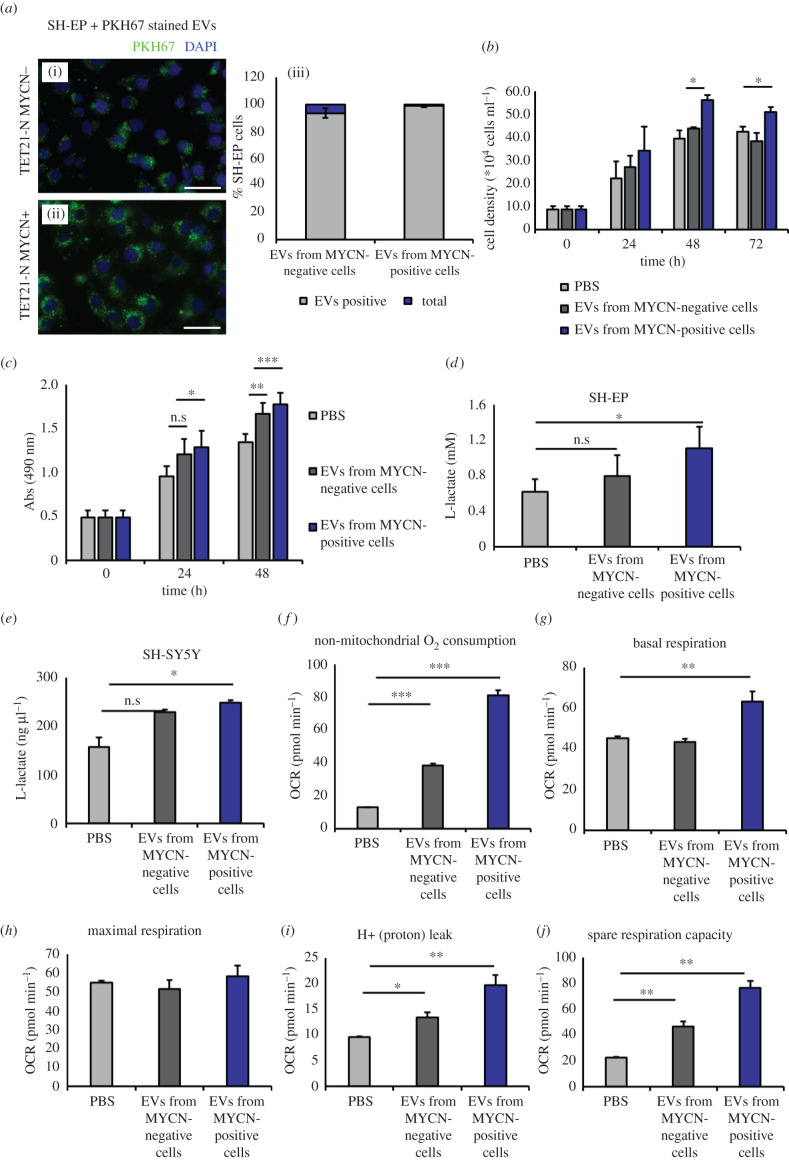
(*Overleaf*.) EVs secreted by MYCN-expressing cells promote glycolysis, respiration, ATP production and proliferation of recipient neuroblastoma cells. (*a*) Fluorescence microscopy of SH-EP cells after 24 h incubation with PKH67-labelled EVs isolated from TET21-N cells expressing or not expressing MYCN. Quantification of the percentages of cells positive to the dye in different conditions is shown on (iii). Error bars indicate mean values ± s.e.m. Scale bar 25 μm. (*b*) SH-EP cells were counted at 1 day intervals after being cultured in the presence or absence of EVs purified from MYCN-positive or -negative TET21-N cells (*n* = 3). (*c*) SH-EP cells were subjected to MTS assays 24 or 48 h after being cultured in the presence or absence of EVs purified from MYCN-positive or -negative TET21-N cells (*n* = 5). (*d*) L-lactate production in SH-EP (*n* = 4) and (*e*) SH-SY5Y (*n* = 3) neuroblastoma cells incubated for 24 h with EVs isolated from TET21-N cells with and without MYCN expression. (*f–j*) Seahorse analysis. MYCN single-copy cell line SH-SY5Y was used as a recipient for EVs purified from TET21-N cells with or without MYCN (*n* = 3). PBS was used as a control (untreated). OCR indicates oxygen consumption rate. Error bars represent mean values ± s.e.m. *p ≤ 0.05, **p ≤ 0.01, ***p ≤ 0.001.

To assess the biological function of EVs in recipient cells, we used the neuroblastoma cell line SH-EP, which is stromal-type, therefore simulating the Schwann stroma surrounding the nests of highly proliferating neuroblasts in tumours. SH-EP cells were incubated with EVs purified from TET21-N neuroblastoma cells in MYCN-off or MYCN-on conditions. Vesicles were also stained with PKH67 and the ability of SH-EP cells to incorporate them was confirmed by fluorescence microscopy. The fluorescent EVs were mainly localized in the cytoplasm of recipient cells (figure 3*a*). To investigate whether the incorporation of EVs had biological consequences, we exposed SH-EP cells to EVs secreted by TET21-N cells in induced or un-induced conditions and measured cell proliferation and metabolic activity. Neuroblastoma cells receiving EVs produced by MYCN-activated cells were stronger inducers of cell proliferation and metabolism compared to EVs produced by MYCN non-expressing cells (figure 3*b,c*). Since glycolytic enzymes were particularly enriched in EVs produced by MYCN-activated cells, we also measured the glycolytic activity of recipient cells. SH-EP cells were incubated with EVs for 24 h and we observed that the secretion of L-lactate was significantly increased in the presence of EVs produced by MYCN-expressing cells (figure 3*d*). This effect was also observed in SH-SY5Y cells, a *MYCN* single-copy cell line (figure 3*e*). Non-mitochondrial oxygen consumption, basal respiration, maximum respiration, spare respiration capacity, proton leak and ATP production were also significantly increased in SH-SY5Y cells upon incubation with EVs secreted from MYCN-positive cells (figure 3*f–j*). Taken together, these experiments suggest that EVs secreted by MYCN-positive cells increase the proliferation rates and metabolic activity of stromal and MYCN single-copy neuroblastoma cells by inducing a Warburg switch.

### 3.4. Neuroblastoma extracellular vesicles induce increased mitotic index and histone H3 (T11) phosphorylation in a pyruvate kinase M2-dependent manner

**Figure 4.**
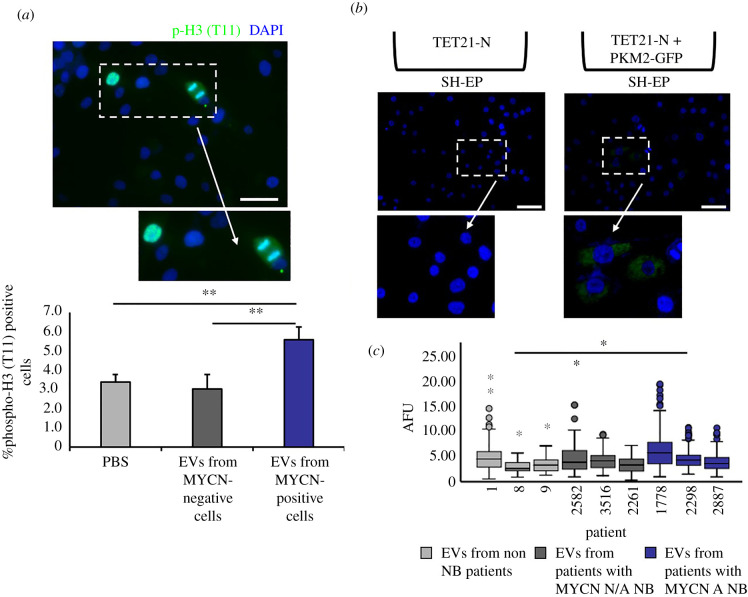
EVs produced by MYCN-expressing neuroblastoma cells induce histone H3 (T11) phosphorylation. (*a*) SH-EP cells were cultured for 24 h in the presence of EVs purified from the supernatants of MYCN-positive or -negative TET21-N cells and stained with an antibody against phospho-histone 3 (T11) (*n* = 4). Scale bar, 50 µm. (*b*) Fluorescence imaging of SH-EP cells after 24 h co-culture with TET21-N cells (uninduced, MYCN-on) overexpressing PKM2-GFP. Scale bar, 75 μm. (*c*) SH-EP cells were treated with EVs isolated from plasmas of neuroblastoma or non-cancer patients and stained with an antibody against phospho-histone 3 (T11). The intensity of the staining expressed as arbitrary fluorescence units (AFU) is displayed in the box plot and the statistical significance of the differences in intensities among groups was verified using an independent-samples *t*-test. Lavene's test was used to verify the assumption of equal variance. N/A NB indicates non-MYCN-amplified neuroblastoma (*n* = 3). Error bars represent mean values ± s.e.m. *p ≤ 0.05, **p ≤ 0.01.

In addition to its role in glycolysis, PKM2 has been shown to cause epigenetic modifications by entering the nucleus and phosphorylating histone H3 on threonine 11. This results in the activation of signalling, transcription of target genes, such as cyclin D1 and c-MYC, and increased cell cycle progression [26]. Thus, we hypothesized that EVs enriched in PKM2 could induce phosphorylation of histone H3 in recipient cells. Indeed, histone H3 (T11) phosphorylation was increased in SH-EP cells incubated with EVs purified from MYCN positive, but not negative, cells (figure 4*a*). To study the role of EV PKM2 in a more physiologically relevant setting, we used a co-culture chamber system in which induced or un-induced TET21-N cells were separated by 0.4 µm pores tissue culture inserts from recipient SH-EP cells (electronic supplementary material, figure S4A). The transfer of EVs from donor to recipient cells was confirmed by transfecting TET21-N cells with a construct expressing a CD63-GFP fusion protein (electronic supplementary material, figure S4B), and observing the GFP signal in the SH-EP recipient cells after 24 h (electronic supplementary material, figure S4C). Using the same methodology, we confirmed that PKM2 is delivered to SH-EP cells co-cultured with TET21-N cells transfected with a construct expressing a PKM2-GFP fusion protein (figure 4*b*). To increase the clinical relevance of these results, we investigated whether EVs isolated from the plasmas of neuroblastoma patients could also induce phosphorylation of histone H3 on threonine 11, focusing on interphase cells. The intensity of histone H3 phosphorylation in interphase SH-EP cells was statistically significantly increased after incubation with EVs purified from patients with *MYCN*-amplified neuroblastomas, compared to patients with non-*MYCN*-amplified tumours or children without cancer, supporting the results obtained with the cell line systems (figure 4*c*).

### 3.5. Vesicular trafficking of pyruvate kinase M2 is required for the increased levels of histone H3 phosphorylation in recipient cells following activation of *MYCN* in donor cells

**Figure 5.**
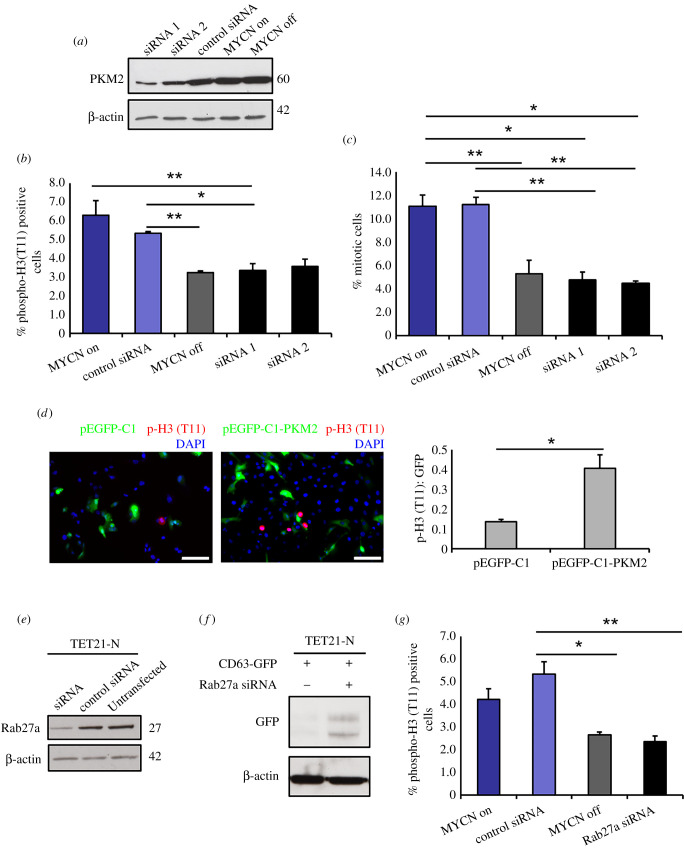
EVs produced by MYCN-expressing neuroblastomas induce mitotic index and histone H3 (T11) phosphorylation in a PKM2-dependent manner. (*a*) Western blot analysis demonstrating the downregulation of PKM2 expression by two different siRNAs. An actin antibody was used as loading control. (*b*) siRNA knock-down of PKM2 in TET21-N donor cells inhibits histone H3 phosphorylation in recipient cells (*n* = 3). (*c*) Mitotic index. The mitotic index is expressed as the percentage of cells in mitosis (prophase, metaphase, anaphase and telophase) over the total number of cells (*n* = 3). (*d*) SH-EP cells were transfected with pEGFP-C1 or pEGFP-C1-PKM2 and stained with an antibody against phospho-histone 3 (T11) after 48 h. The number of transfected and phospho-histone H3 (T11) positive cells was quantified in each condition (*n* = 3). Scale bar, 100 μm. (*e*) Western blot analysis demonstrating downregulation of Rab27a expression by siRNA. An actin antibody was used as loading control. (*f*) Western blot analysis of CD63-GFP overexpressing cells in the presence or absence of the anti-Rab27a siRNA. An actin antibody was used as loading control. (*g*) siRNA knock-down of Rab27a in TET21-N donor cells inhibits histone H3 phosphorylation in recipient cells (*n* = 3). Error bars represent mean values ± s.e.m. *p ≤ 0.05, **p ≤ 0.01.

To link the biological effects observed in recipient SH-EP cells to the transfer of vesicular PKM2, we used two independent siRNAs that caused a reduction of PKM2 expression in donor TET21 N cells (figure 5*a*). Histone H3 phosphorylation in SH-EP cells was significantly downregulated in the presence of the PKM2 siRNAs or when the expression of MYCN was turned off in donor cells, strongly suggesting that the transfer of PKM2 is essential to induce histone H3 phosphorylation in recipient cells (figure 5*b*). Since histone H3 phosphorylation is tightly linked to mitosis, we calculated the mitotic index of SH-EP cells cultured in the different conditions and verified that the mitotic index was high when MYCN was activated, but reduced to baseline levels when MYCN was switched off or PKM2 was inhibited by siRNAs in donor cells (figure 5*c*). To further demonstrate that the increase of histone H3 phosphorylation was caused by the transfer of PKM2 in the recipient cells, we transfected SH-EP cells with plasmids encoding GFP or PKM2-GFP, and 48 h later, we assessed phospho-histone H3 (T11). There was a significant increase of histone H3 (T11) phosphorylation in cells transfected with PKM2-GFP compared to GFP control transfection, demonstrating the direct role of the kinase in this effect (figure 5*d*). To ascertain whether the induction of histone H3 phosphorylation was mediated by exosomes or exosome-like EVs, we used siRNAs to downregulate Rab27a, which were previously shown to inhibit exosome secretion [27], in TET21-N cells (figure 5*e*). Rab27a silencing caused intracellular accumulation of the tetraspanin and EV marker CD63 fused to GFP, suggesting that exosomes were not released in the extracellular space in this setting (figure 5*f*). Silencing Rab27a phenocopied the loss of PKM2 expression in co-culture experiments, demonstrating that vesicular transfer of PKM2 is required to induce histone H3 phosphorylation in recipient cells (figure 5*g*).

### 3.6. MYCN-regulated extracellular vesicles activate c-MYC expression in neuroblastoma stromal cells

**Figure 6.**
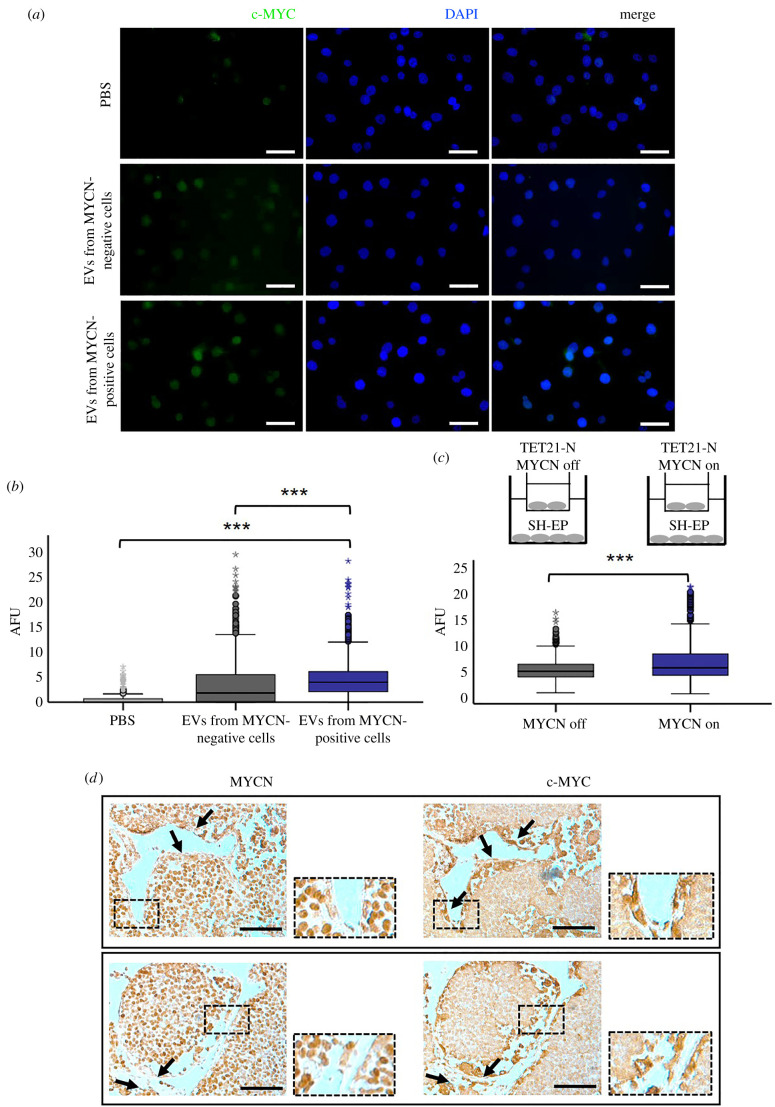
MYCN-regulated EVs activate c-MYC expression in recipient neuroblastoma cells. (*a*) Immunofluorescence analysis with an antibody against c-MYC was carried out in SH-EP cells co-cultured for 24 h with EVs purified from supernatants of TET21-N cells expressing or non-expressing MYCN. PBS was used as a control. Scale bar, 50 µm. (*b*) Quantification of the experiment shown in (*a*) (*n* = 3). (*c*) Quantification of c-MYC immunofluorescence staining in SH-EP cells after 24 h co-culture with TET21-N cells expressing or non-expressing MYCN (*n* = 3). ***p ≤ 0.001. (*d*) Stromal cells surrounding MYCN-positive tumour nests express c-MYC. Neuroblastoma tumours developing in TH-MYCN mice were fixed, embedded in paraffin and serial sections stained with MYCN or c-MYC antibodies. Arrows indicate stromal cells negative for MYCN but expressing c-MYC. Scale bar, 75 µm.

**Figure 7.**
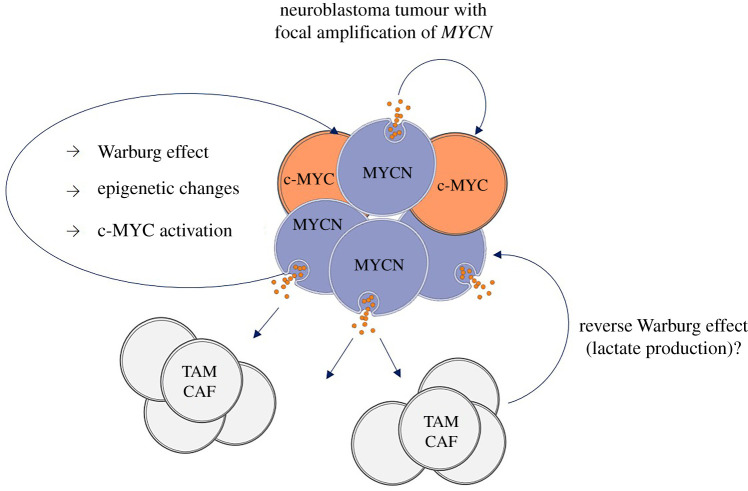
Cartoon illustrating a hypothetical model of neuroblastoma vesicles induced regulation of the tumour microenvironment. *MYCN*-amplified cells spread vesicles loaded with proteins that mediate oncogenic signals in MYCN non-amplified parts of the cancer and cells of the tumour microenvironment. TAM = tumour-associated macrophage. CAF = cancer-associated fibroblast.

A consequence of epigenetic modification of histones induced by nuclear PKM2 is activation of *c-MYC* [26]. We therefore quantified c-MYC expression by immunofluorescence analysis in SH-EP cells exposed to purified EVs or co-cultured in transwell chambers with TET21-N cells in induced or un-induced conditions. EVs purified from TET21-N cells with activated MYCN induced higher expression of c-MYC than EVs purified from cells in which MYCN expression has been switched off (figure 6*a,b*). Similarly, c-MYC expression was significantly increased in SH-EP cells co-cultured with TET21-N cells with MYCN switched on, compared to the condition in which MYCN was switched off (figure 6*c*). In a syngeneic mouse model of neuroblastoma (TH-MYCN), c-MYC expression is detected in stromal cells surrounding nests of MYCN-positive neuroblastomas (figure 6*d*). Overall, these experiments corroborate the hypothesis that the transfer of EVs from MYCN-positive cells induces activation of c-MYC in recipient stromal cells. A cartoon illustrating how the MYCN-regulated vesicles could change the tumour microenvironment is shown in figure 7.

## 4. Discussion

The role of MYC as a regulator of the tumour microenvironment, in addition to its intrinsic effects on cancer cells, is emerging. For example, c-MYC has been shown to alter immune cells metabolism and the cancer microenvironment by supporting the expression of HIF-1 and secretion of VEGF in tumour cells [28]. In neuroblastomas, *MYCN* amplification is conducive to reduce immune infiltration [29,30] and neuroblastoma cells have been shown to secrete macrovesicles able to stimulate the production of pro-tumourigenic signals by bone marrow stromal cells [31]. These studies prompted us to investigate the hypothesis that MYCN could exert non-cell autonomous effects by regulating EVs.

Using mass spectrometry analysis, we have identified MYCN-regulated vesicular proteins mainly clustered in three functional groups: ribosomal proteins, proteins involved in ECM interactions and glycolytic enzymes. We focused our attention on the latter group since it is well established that MYC proteins are regulators of cell metabolism and the Warburg effect. The regulation of metabolism is critical for the function of cellular processes and aggressive cancer cells are able to adapt their metabolism in a way that sustains tumour growth and metastatic dissemination. While most differentiated cells use mitochondrial oxidative phosphorylation as their main source of energy production, cancer cells shift towards aerobic glycolysis by increasing their glucose uptake and metabolizing it to lactate in aerobic conditions, a process known as the Warburg effect [32,33]. A number of enzymes implicated in the glycolytic pathway have been shown to be overexpressed in multiple cancers [34]. The M2 splice isoform of pyruvate kinase is increased in cancer with respect to the M1 variant, mainly found in normal tissues, and contributes to the metabolic adaptations required in tumourigenesis [35,36]. PKM2 has been shown to be modified by acetylation by p300, which transforms it from a cytoplasmic metabolic kinase to a nuclear protein kinase [37,38]. In glioma, PKM2 mediates phosphorylation of histone H3 on threonine 11, resulting in the transcriptional activation of c-MYC, cyclin D1 and tumour progression [26]. In addition, PKM2 regulates chromosome segregation and mitosis in cancer by interacting with the spindle checkpoint protein Bub3 [39].

MYC regulates lactate production, glutaminolysis, and increase protein translation by promoting ribosome biogenesis [40]. MYCN is also involved in metabolic reprogramming of neuroblastoma cells by enhancing glycolytic metabolism [41]. Among the glycolytic enzymes enriched in the EVs of MYCN-expressing neuroblastoma cells, we identified hexokinase II and PKM2. Notably, we detected the presence of hexokinase II and PKM2 in EVs circulating in the bloodstream of neuroblastoma patients and more frequently in those bearing *MYCN*-amplified tumours. The glycolytic enzymes were undetectable in the EVs of non-cancer patients, supporting the hypothesis that they are of cancer origin. Given that the expression of *PKM* and *HK2* in neuroblastoma samples is strong predictors of negative clinical outcome, analysis of the protein content of circulating EVs could be used as a prognostic indicator and a non-invasive method for stratification of neuroblastoma patients.

*MYCN*-amplified cells are more sensitive to the depletion of PKM2 than *MYCN* non-amplified cells, suggesting oncogenic addiction [42]. This is because the PKM2 isoform is enriched in *MYCN-*amplified neuroblastoma cells due to the ability of the MYC transcription factors to transactivate the splicing factors *PTBP1* and *HNRNPA* [42,43]. It is tempting to speculate that the transfer of oncogenic kinases via EVs operated by MYC mutant cells could contribute to the aggressive behaviour of the overall tumour mass by promoting the metabolic activity of cancer or stromal cells that do not carry oncogenic mutations. PKM2-enriched neuroblastoma EVs have the potential to modify epigenetically recipient cells by enhancing histone H3 phosphorylation. This would permanently fix transient oncogenic signals, perhaps explaining why *MYCN*-amplified tumours can revert to focally amplified, and focally amplified to non-amplified, during the evolution of the disease [7].

A limitation of this study is that we have not examined the role of nucleic acids in neuroblastoma EVs, although functional microRNAs have been previously detected in EVs secreted by neuroblastoma cell lines or immune cells infiltrating neuroblastomas [44,45]. It is not possible to exclude that the transfer of MYCN-regulated microRNAs might have contributed to some of the biological effects observed in our study. Indeed, two research groups have independently shown that microRNAs belonging to the 17–92 cluster are enriched in vesicles secreted by *MYCN*-amplified cells and their transfer stimulate proliferation and migration of recipient, *MYCN* non-amplified cells [45,46]. However, RNAi inhibition of PKM2 in *MYCN-*expressing donor cells completely reversed the increase in histone H3 phosphorylation in recipient *MYCN* single-copy cells, suggesting that this effect is regulated specifically by protein transfer. In conclusion, the results of our study suggest that *MYCN*-amplified neuroblastomas might promote the spreading of oncogenic kinases and other biologically active proteins to the tumour microenvironment and remote body locations.

## Data accessibility

The mass spectrometry proteomics data have been deposited to the ProteomeXchange Consortium via the PRIDE repository at https://www.ebi.ac.uk/pride/.

The data are provided in the electronic supplementary material [47].
